# The Relationship between Vegetable Intake and Weight Outcomes: A Systematic Review of Cohort Studies

**DOI:** 10.3390/nu10111626

**Published:** 2018-11-02

**Authors:** Monica Nour, Sarah Alice Lutze, Amanda Grech, Margaret Allman-Farinelli

**Affiliations:** Charles Perkins Centre, School of Life and Environmental Sciences, The University of Sydney, Sydney, NSW 2006, Australia; salu7152@uni.sydney.edu.au (S.A.L.); agre3682@uni.sydney.edu.au (A.G.); margaret.allman-farinelli@sydney.edu.au (M.A.-F.)

**Keywords:** vegetables, weight gain, obesity, adults

## Abstract

The relationship between vegetable consumption and measures of adiposity was assessed in cohort studies. Seven databases were searched from inception until October 2018. The quality of individual studies was assessed using the Joanna Briggs Institute Critical Appraisal of Cohort Studies tool. The Grading of Recommendations Assessment, Development and Evaluation (GRADE) system was applied to determine the quality of the body of evidence. Ten studies were included. Six measured change in vegetable intake over time. Two showed that increasing vegetable consumption resulted in weight loss of 0.09–0.1 kg over four years (*p* < 0.001). Increased vegetable intake was also associated with a reduced risk of weight gain and overweight or obesity (Odds ratios (ORs) ranged from 0.18 to 0.88) in other studies. Four studies measured vegetable intake at the baseline only. One showed that intakes >4 servings/day reduced the risk of weight gain (OR 0.27 (95% confidence interval (CI) 0.08–0.99) and another found an inverse association with waist circumference in women (−0.36 cm per vegetable serving/day). This review provides moderate quality evidence for an inverse association between vegetable intake and weight-related outcomes in adults. When these findings are coupled with no apparent harm from vegetable consumption, the evidence-base can be used with acceptable confidence to guide practice and policy.

## 1. Introduction

The global prevalence of overweight and obesity has increased substantially since 1980 [1]. In 2016, the World Health Organization (WHO) estimated that 52% of adults were classified as overweight or obese [2]. Being above a healthy weight is a major risk factor for the development of diet-related chronic diseases, such as cardiovascular disease, stroke, type 2 diabetes, and certain cancers [1]. Nutrition and more specifically, fruit and vegetable intake, is a key modifiable factor for the prevention of chronic diseases [3,4,5]. As such, these nutrient dense, fibre-rich foods have been promoted worldwide as fundamental components of a healthy diet [6,7,8]. A minimum intake of 400 g of fruits and non-starchy vegetables per day has been recommended by WHO to reduce the global burden of noncommunicable diseases [9,10,11]. A serving of fruits or vegetables is equivalent to 80 g, and thus a minimum of five servings should be eaten daily [12,13].

It is proposed that a generous intake of fruit and vegetables may also assist in weight management because they are low in energy but high in fibre and water to produce a satiating effect. It is postulated that the satiating properties of fruit and vegetables are the mechanism whereby reduced consumption of energy-dense nutrient-poor foods occurs, lowering the overall calorie intake [14,15]. There have been several clinical trials of both energy-controlled and more liberal diets enriched with fruits and vegetables to promote weight loss [16,17,18,19]. A meta-analysis of experimental studies concluded that the impact of increasing fruit and vegetable intake is small and insignificant when advice is given without instruction to restrict energy from other foods [19]. Conversely, a review of experimental studies which explored the benefits of vegetables specifically within a healthy balanced diet, compared to usual dietary intake in the control group, identified five studies where significant weight loss was observed [20]. This suggests that simply increasing a given food such as vegetables may not result in weight loss without due consideration of the total diet.

The DASH (Dietary Approaches to Stop Hypertension) and Mediterranean diets provide further evidence for the role of vegetables in weight management in the context of a complete diet. Such diets encourage high intakes of fruits and non-starchy vegetables, but limit other energy-dense nutrient-poor foods. Research documenting the impact of these dietary patterns has indicated their effectiveness in producing significant weight loss in the short-term [21,22,23]. While there is some evidence from randomized controlled trials indicating that vegetables play a role in weight management over short periods, inconsistencies in results are found [20].

Longitudinal studies explore food intake in larger populations who are free-living and self-select their diet. Thus, these observational studies are more useful to provide evidence about long-term impacts of diets with high and low fruit and vegetable intakes on weight status. Reviews that summarise the collective impact of longitudinal/epidemiological studies have concluded that there is weak or limited evidence for a protective relationship between the intake of fruits and vegetables and weight-related outcomes [24,25]. Fruits and vegetables have typically been studied together. While fruits and vegetables have similar properties, there are differences in their nutritional composition and the manner in which they are incorporated into the diet [26]. Non-starchy vegetables usually contain more water and fibre than fruits that are higher in sugar (fructose). Fruits are more likely to be eaten raw, as a snack, or as a dessert, whereas vegetables are usually consumed cooked in mixed dishes [26] or with additional meal components, and may thereby impact on other foods eaten during a meal. Therefore, it is possible that fruits and vegetables have differing effects on weight. The impact of fruit on weight-related outcomes has been studied, with evidence indicating an inverse relationship [27,28]. The association between vegetables alone and weight outcomes has not been studied as thoroughly. This provides a strong rationale for exploring the independent role of vegetables in weight management. The most recent meta-analysis of cohort studies investigating vegetables independently of fruit was published in 2015 and found no significant association with weight, but a reduced risk of adiposity with increasing vegetable intake [29]. 

The aim of this systematic review was to update the evidence base on the relationship between vegetable intake and change in weight-related outcomes among adults using prospective cohort studies.

## 2. Materials and Methods

The Preferred Reporting Items for Systematic Reviews and Meta-Analyses (PRISMA) framework was used to guide the systematic review process and reporting of outcomes [30]. 

### 2.1. Search Strategy

Seven electronic databases were searched (Cochrane Library, Medline, PreMedline, Cinahl, Science Direct, Scopus, and Embase). The last search was conducted on the 8th of October 2018. Combinations, synonyms, and truncations of “vegetable” and “body weight” or “weight gain/increase/change/maintenance” or “overweight” or “obesity” or “Body Mass Index” (BMI) and “cohort” or “longitudinal” were used. Medical Subject Headings (MeSH) terms were also utilised in Medline to expand the search. For example, “cohort” included the terms “incidence” and “follow up”. An example of the full search strategy conducted in Medline is outlined in Table 1. The grey literature was searched for documents such as government or academic reports and conference abstracts relevant to the research question. Reference lists were also searched for additional studies. 

### 2.2. Eligibility Criteria

Observational cohort studies with at least one follow-up that explored the association between vegetable intake and weight-related outcomes, such as body weight, BMI, waist circumference, or adiposity, were included. Studies examined adult populations (adolescents were included if followed into adulthood) free of disease or illness that impacts on weight or the cognitive ability to modify one’s own dietary intake. The search was restricted to papers written in English; however, no limiting time-frame was applied to date of publication. 

### 2.3. Study Selection

**Figure 1:**
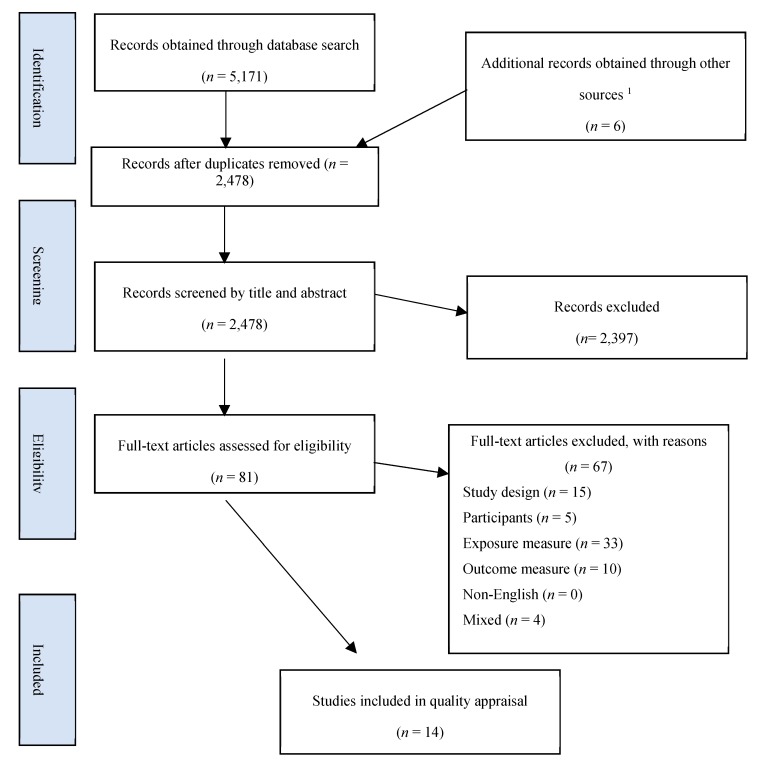
Flowchart of literature search and screening for selection of cohort studies exploring the impact of vegetables on anthropometric outcomes. ^1^ Other sources included a Google search, a hand search of reference lists of relevant systematic reviews and included studies, GRADE; Grading of Recommendations Assessment, Development and Evaluation.

All studies retrieved from database searches were exported to Endnote X8 citation management software (Thomson Reuters, Toronto, Canada). Figure 1 displays a flowchart of the process of selection. After the removal of duplicates, titles and abstracts were assessed in accordance with the eligibility criteria. The studies which were deemed potentially relevant to the review were downloaded as a full text and independently reviewed for eligibility by two reviewers. If discrepancies in judgement arose, a third reviewer was consulted. 

### 2.4. Data Extraction

A table was created to collate the data. Guided by the PRISMA statement for reporting systematic reviews [30], the key components extracted were; study design, participant characteristics, exclusion/inclusion criteria, duration of study, retention of participants, and study results. Additional items related to study quality, such as adjustment for confounders, statistical tests used, and the exposure and outcome measurement methods, were added to the table to include fields needed to assess study quality [31]. For each study, two reviewers extracted the data.

### 2.5. Quality and Risk of Bias Assessment for Individual Studies

The study quality and presence of biases were determined using the Joanna Briggs Institute Critical Appraisal Checklist for Cohort Studies [31] (Table 2). Three authors completed quality assessments for the 14 studies deemed eligible for inclusion. Each study was assessed twice. The tool is comprised of 11 questions regarding the study design, with the option to answer ‘yes’, indicating higher quality; ‘no’, indicating poor quality; or ‘unclear’. The questions address the selection bias, validity, and reliability of methods used for the measurement of exposure and outcomes, confounding, length of study, reverse causality, and appropriateness of statistical analysis, and adjustment for key confounders. Appraisal using this tool allowed authors to either ‘include’ or ‘exclude’ studies based on overall quality. If a study had ≥3 ‘no’ or ’unclear’ quality categories, then it was excluded from the analysis. Any discrepancies in judgements regarding inclusion were resolved through discussion. The outcomes of the appraisal process conducted by the two reviewers are presented as a supplementary table (Table S1).

### 2.6. GRADE Assessment for Quality of Overall Body of Evidence

Two reviewers determined the overall quality of the body of evidence using the GRADE system (Grading of Recommendations Assessment, Development and Evaluation) [32]. This process is important in determining the degree of confidence placed on the overall findings in guiding practice recommendations. The system assesses the overall evidence against five categories that increase or decrease confidence in the results. These categories are; limitations in study designs (risk of bias, consistency of the reported results, directness of the evidence (including how directly the studies capture the study population and outcomes of interest)), the precision of outcomes, and whether publication bias may be present.

## 3. Results

### 3.1. Study Selection

As shown in Figure 1, a total of 5172 articles were obtained by database searching. A further six studies were identified through hand searching reference lists and the grey literature. After the removal of duplicates, 2478 records were screened by title and abstract, of which 2397 were excluded, leaving 81 papers for full-text assessment. Fourteen studies [33,34,35,36,37,38,39,40,41,42,43,44,45,46] were found to meet eligibility criteria and were included in this review for quality appraisal.

### 3.2. Quality and Risk of Bias Assessment Using the Joanna Briggs Institute (JBI) Critical Appraisal Checklist for Cohort Studies

As shown in Table 2, four eligible studies received a poor quality rating, as determined by the JBI critical appraisal tool. Data was extracted from these studies and considered, but findings were excluded from the overall body of evidence. The main reasons for the exclusion of these studies were the use of un-validated methods for measurement of the exposure or the outcome, failure to use strategies for dealing with confounders, and poor statistical analysis (such as not controlling for energy intake or adjusting for incomplete data) [34,38,39] (Table 2). Item number six of the checklist was not applicable to the research question, as it was not important for participants to be free of the outcome at baseline. All studies had a sufficient follow-up time for outcomes to occur. However, only two of the included studies provided a specific explanation of strategies used to address incomplete follow-up [33,40]. Four studies did not adjust for energy intake, a key confounding factor to account for when exploring dietary predictors of weight gain [34,35,39,46]. Among the ten included studies, vegetable intake was measured using different validated food frequency questionnaires (FFQ) or short dietary questionnaires (Table 3). Anthropometric outcomes were collected through validated self-report questionnaires or measured by trained technicians (Table 3). 

### 3.3. Summary of Included Studies

Table 4 presents the characteristics of the 14 eligible studies. Of the ten included studies, five were conducted in the USA [33,37,40,41,42]. One study used the European Prospective Investigation into Cancer and Nutrition EPIC cohort (participants from 10 different European countries) [44] and the remaining used cohorts from Spain [45], Japan [43], Iran [35], and Denmark [36]. Two studies focused on women only [37,42] and the remaining were both genders. One study only included individuals who were of normal BMI (18.5 to <25 kg/m^2^) at baseline [42]. The age of participants ranged from 15 years [41] to 80 years [45]. The duration of follow-up ranged from one year [43] to 20 years [40], and two studies had multiple follow-up intervals of four years [33,40]. Sample sizes ranged from 206 [45] to 120,844 [40]. There was an overlap of participants as two studies included three of the same cohorts [33,40], including the Nurses’ Health Study (NHS), Nurses’ Health Study II (NHS II), and Health Professionals Follow-Up study (HPFS); whilst one other study also used the NHS cohort [37]. The retention rate for the majority of studies fell between 81–96%, with one study reporting a considerably lower retention of 66.4% [41] and one indicating a response rate of 51% [45].

### 3.4. Measurement of Exposure

Vegetable intake data was collected using validated questionnaires in all the included studies at baseline. However, four studies did not measure vegetable consumption at follow-up [36,42,43,44] and one used a self-report question that was not validated to determine a change in intake after the 10-year study period [45] (Table 3). Of the four excluded studies, only the small study by Butler and colleagues used a validated measure of vegetable intake [34]. Intake was reported by a majority of studies as servings [33,35,37,40,41,42] or grams per day [44,45]. One study recorded change in intake as grams/1000 kcal/day [43], with another as change per 60 kcal/day [36] (Table 3). For this later study, we converted the units to 1 serving/day to allow comparison across studies. This change was made using the assumption that the median kilojoule value per vegetable serving is 60 kcal (250 kJ) [8]. Three studies separated potatoes from vegetable intake in their findings [33,36,40]; this was considered when interpreting results.

### 3.5. Measurement of Outcome

The outcome measures were weight change [33,35,40,43,44,45], risk of becoming overweight or obese [37,41,42], and change in waist circumference [36]. For the included studies, outcomes were measured by trained technicians [35,43,44,45]; self-reported [33,37,40,41,42]; or in the case of Halkjaer et al., conducted by trained technicians at baseline and self-reported at follow-up [36]. Self-reported weight measures were validated by an objective measurement of outcome in subsamples of the cohorts. Of the four excluded studies, two used non-validated measures of weight outcomes [38,39].

### 3.6. Association between Vegetable Intake and Anthropometric Outcomes

The relationships between vegetable intake and anthropometric measures reported in the fourteen eligible studies are summarised in Table 5. Wherever possible, results extracted and summarised are based on the association between one vegetable serving and anthropometric outcomes. The results of studies that measured vegetable intake at baseline only [36,42,43,44] are presented after those assessing intake over time. Five studies reported intake according to quartiles/quintiles [37,38,42,43,45].

### 3.7. Associations from Studies with Change in Vegetable Intake over Time

The two studies that used the same three cohorts (NHS, NHS II, and HPFS) both concluded that vegetable intake was inversely associated with weight (Table 5) [33,40]. However, one study [40] excluded participants with obesity. The study that included individuals of all weight statuses found that a higher vegetable intake was a greater predictor of weight loss for overweight individuals than it was for those of a healthy-weight (*p* = 0.03) [33]. They also found that unlike non-starchy vegetables, potatoes (baked, boiled, or mashed) were positively correlated with weight gain. Another study used data from the NHS cohort [37], reporting that women with the largest increase in vegetable consumption had the lowest risk of obesity (*p* = 0.0002), with intakes of approximately one serving daily decreasing the risk by 15% (Table 5). Women from the Iranian Lipid and Glucose study who decreased their vegetable intake were less likely to report weight loss [35]. The association was not significant in males. In contrast, among the youth in the Project EAT cohort, increased vegetable intake was protective against the incidence of overweight in males, but not females [41]. Finally, a study conducted in Spain found the strongest relationship between vegetables and weight gain prevention. The authors reported that over 10 years, those who consumed the highest vegetable quantities (>333 g/day) at baseline had the lowest risk of gaining >3.4 kg compared to those who consumed the least (<166 g/day) (OR: 0.18; 95% CI 0.05 to 0.66). Based on the WHO definition of a serving of vegetables as 80 g [16], these results suggest that the consumption of four servings daily reduces the risk of weight gain by 82% compared to only two serves consumed per day. However, our confidence in the strength of this relationship is limited as the analysis was based on baseline intakes adjusted for self-reported change in vegetable intake measured as “yes/no”. A forest plot has been drawn to assist with comparison of the effect sizes for these studies (see supplementary Figure S1).

### 3.8. Associations from Studies with Baseline Vegetable Intake

Four studies assessed vegetable intake at baseline only [36,42,43,44]. Halkjaer et al. found an inverse association between vegetable intake and waist circumference among women. Potatoes were shown to be positively associated with waist circumference in women, but no significant relationships were found between intakes of vegetables and waist circumference in men (Table 5) [36]. Vergnaud et al. found that after controlling for variations in dietary measurement between the European centres comprising the EPIC cohort, there were no significant associations between vegetable intake and change in weight over 10 years [44]. Rautiainen et al. concluded that the risk of becoming overweight or obese for women is not significantly related to vegetable intake at baseline [42]. Finally, the study exploring changes in weight in a cohort of Japanese factory workers, found that vegetables reduce the risk of gaining ≥3 kg weight in a single year by 73% [43] (Table 5).

### 3.9. Association from Excluded Studies

Of the four studies which were downgraded due to a poor study quality, two found significant associations with anthropometric outcomes. Butler and colleagues showed that within the 20-week observation period, a decreased vegetable intake was associated with increases in weight, BMI, and fat mass [34]. Another study found that consumers within the highest intake quintile had reductions in BMI and the risk of weight gain at the waist compared to those in the lowest quintile [38]. 

### 3.10. Grading of Recommendations Assessment, Development and Evaluation (GRADE) Assessment for Quality of Overall Body of Evidence

There were some limitations among the ten reviewed cohort studies that reduced our confidence in the quality of the overall body of evidence. These limitations are listed below and were considered by two independent reviewers who collaborated to determine the overall quality of the current evidence to be moderated (Table 6).

#### 3.10.1. Study Limitations

The removal of studies considered to have a poor study design or high risk of bias, as determined using the JBI tool, strengthened the quality of included studies. However, five of the reviewed studies failed to measure changes in intake over time using valid tools [36,42,43,44,45]. Thus, studies that have explored associations using baseline data only are not considered strong sources of evidence.

#### 3.10.2. Inconsistencies

The body of evidence was consistently in favour of the inverse relationship between vegetables and weight-related outcomes. Only two of the ten studies found no significant association, with the remaining eight studies indicating that increasing vegetable intakes reduces the risk of weight gain or of becoming overweight or obese. 

#### 3.10.3. Directness

The included papers directly address the population of interest. However, as some of the studies did not explore the change in vegetable intake over time (associations based on baseline intake), the measurement of exposure is indirect.

#### 3.10.4. Precision

Sample size varied from 206 [45] to 120,844 and totalled 796,069, which is considered more than sufficient. While three studies had an overlap in the populations included in the analyses, the cohorts were large enough to be considered as strong evidence on their own. 

#### 3.10.5. Publication Bias

While lack of publication bias cannot be assumed, the authors comprehensively searched the grey literature and reference lists of extracted studies. Additionally, papers with both significant and insignificant outcomes have been included.

## 4. Discussion

The six reviewed studies that explored change in vegetable consumption over time found a favourable relationship between increasing vegetable intake and weight-related outcomes outside of controlled study environments. Three of the largest studies contributing to this evidence used overlapping cohorts from surveys conducted with health professionals [33,37,40]. However, given the large size of these cohorts, and their robust study design, they provide good quality evidence when considered alone. The remaining studies assessed intake at baseline only or were deemed to be of a very poor quality, and so the impact of the total body of evidence presented is interpreted with caution.

This review found differences in the impact of vegetables on weight gain versus weight loss. The studies which explored the risk of weight gain according to quantiles of vegetable intake [37,38,43,45] revealed that the relationship is dose dependent, such that higher intakes were associated with the lowest risks of weight gain. The most substantial risk reduction was observed in a study of Spanish adults, which showed that those consuming four or more vegetable servings per day over a 10-year period had an 82% reduced risk of gaining more than 3.4 kg. This evidence suggests that diets that more closely adhere to the recommended daily vegetable target may reduce the risk of weight gain in the longer term. 

With regards to weight loss, however, the impact appears small. Together, the papers using the NHS, NHSII, and HPS cohorts reported an inverse association between vegetable intake and weight. They showed that each extra vegetable serving per day resulted in weight loss between 0.09–0.1 kg over four years [33,40]. These small improvements indicate that vegetables are important for weight maintenance, but may not necessarily promote weight loss.

Two studies showed gender specific associations between vegetable intake and weight change [35,41]. Among the Iranian cohort, a decreased vegetable intake had no significant effect on the likelihood of weight loss in men, while women were 56% less likely to lose weight. In contrast, the study which tracked adolescents for 10 years showed that each additional daily serving of vegetables reduced the risk of becoming overweight in males but not females [41]. Previous literature has found that adolescent females are more likely to under report weight, over report healthy foods, and participate in dieting [65]. No physiological mechanism whereby one gender would be more influenced by vegetable intakes is readily apparent, but this warrants further investigation.

A differential effect was found according to baseline weight status. The reduced risk of overweight/obesity with higher vegetable intakes was found to be greater for individuals above a healthy weight. This pattern of additional benefit among individuals with a higher weight has also been observed in a review of experimental trials promoting fruits and vegetables to reduce adiposity [24]. Ledoux suggests this phenomena may be related to poorer dietary patterns among overweight individuals at baseline compared to those of a healthy weight, providing greater opportunity for improving diet quality over time [24]. 

The eight cohort studies identified here that did not validly measure change in vegetable consumption over time with weight outcomes, or were downgraded due to poor quality, do provide some further evidence to support the role of vegetables in weight management. Two studies showed that higher intakes of vegetables reduce the risk of weight gain >3.4 and 3 kg, by 82% and 73%, respectively [43,45]. One study found that with each vegetable serving, the waist circumference of women is reduced by 0.36 cm over a period of approximately five years (no association in males). Two studies of a poorer quality showed that a lower vegetable intake was not favourable for anthropometric outcomes, including weight, BMI, fat mass, or weight gain at the waist [34,38]. The remaining four studies found no significant associations [39,42,44,46].

Studies that explored the relationship between potato intake and weight-related outcomes separately to other vegetables found that the intake of these starchy vegetables are positively correlated with weight and waist circumference [33,36,40]. Although potatoes are nutrient dense, providing a good source of potassium, vitamin B6, and some plant protein, they have a higher energy content from starch and present a higher glycemic load than non-starchy vegetables. Their positive association with weight-related outcomes may result from the energy and fat deposition promoted by a higher insulin response [66]. Non-starchy vegetables provide more food (in grams) per kilojoule due to their higher water and fibre content, and so are more satiating. It is therefore suggested that all future cohort studies explore the impact of potatoes separately to non-starchy vegetables. 

Collectively, the evidence from studies with valid measures of vegetable intake over time suggests an association that supports the outcomes observed in experimental trials where higher vegetable consumption protected against weight gain, assisted with weight maintenance, or supported weight loss [16,18,67,68]. The extracted body of evidence contained five studies that measured vegetable intake at baseline only. Consumption levels reported at one time point are unlikely to remain the same, with evidence indicating that diet quality improves over time [69,70]. Thus, there remains an opportunity for epidemiologists to develop well-designed longitudinal cohort studies that specifically measure the change in vegetable consumption over time (using valid tools). Furthermore, it is recommended that future studies explore the association of potatoes separately from other vegetables or make the study data available to researchers so further analysis can be conducted to determine the independent relationship between potatoes and weight-related outcomes.

Previous reviews have confirmed the inverse relationship between weight/adiposity [27,28,29] and fruit intake, but the association with vegetable consumption remains questionable. The most recent meta-analysis of cohort studies investigating this relationship was published in 2015. The highest quintiles of vegetable intake were found to be associated with a 17% reduced risk of adiposity [29]. The meta-analysis found no significant association between vegetable intake and weight when the studies by Mozaffarian et al. and Vergnaud et al. were pooled together. The latter study only measured vegetable intake at baseline. Our review included two new studies in favour of an inverse association between vegetables and weight, providing an updated summary of the evidence on vegetables and weight-related outcomes. The most significant strength of this review is that it provides the first summary of cohort studies on the association of weight outcomes and vegetable intake specifically, without reporting on the impact together with fruit.

One of the major limitations of our review, however, is the overlapping cohort population in three of the included studies and the heterogeneity of anthropometric outcomes used to measure impact on weight. These factors prevented the pooling of data using meta-analysis. While the studied cohorts collectively form a sample large enough to be representative of the western world, average intakes within the population are known to be inadequate [71]. Thus, the effect of vegetable consumption at the recommended daily amount on weight is yet to be determined.

In conclusion, the majority of studies reviewed support an inverse relationship between vegetables and weight-related outcomes in free-living individuals. Public health practitioners and those responsible for the development of evidence-based national guidelines must draw upon the combined findings of experimental and cohort studies to inform recommendations to increase vegetable intake for weight management. The apparent lack of harmful effects of vegetable consumption in the general population, as well as their independent role in preventing other non-communicable diseases such as cardiovascular disease, stroke, and some cancers [4,5] gives logic to the recommendation. Despite these benefits, consumption levels remain well below those recommended worldwide [71] and so increasing intakes should remain a focus in public health efforts to reduce the global burden of chronic disease.

## Figures and Tables

**Table 1:** Electronic database search strategy: Medline (via Web of Science).

Search No.	Search Statement	No. of Citations Retrieved
**1**	((((MESH MAJOR TOPIC: exp: (((((body weight) OR Body Weight Maintenance) OR Body Weight) OR Body Weight Changes) OR Weight Gain) OR Weight Loss) OR MESH MAJOR TOPIC: exp: (Obesity)) OR MESH MAJOR TOPIC: exp: (overweight)) OR TOPIC: (increase NEAR/2 weight)) OR TOPIC: (change NEAR/2 weight)) OR ((TOPIC: (maintenance NEAR/2 weight AND LANGUAGE: (English)) AND SPECIES: (Humans))	438,097
**2**	(MESH MAJOR TOPIC: (vegetable *) AND LANGUAGE: (English)) AND SPECIES: (Humans))	3566
**3**	(MESH MAJOR TOPIC: exp: (Cohort Studies OR Longitudinal Studies) AND LANGUAGE: (English)) AND SPECIES: (Humans))	1,428,860
**4**	#3 AND #2 AND #1	39

* search term as major focus of articles; #, search number.

**Table 2:** Quality assessment using the Joanna Briggs Institute (JBI) Critical Appraisal Checklist for Cohort Studies.

JBI Checklist no. Study	Joanna Briggs Institute Critical Appraisal Checklist for Cohort Studies											
	1. Two Groups Similar and Recruited from the Same Population?	2. Were the Exposures Measured Similarly to Assign People to Both Exposed and Unexposed Groups?	3. Was the Exposure Measured in a Valid and Reliable Way?	4. Were Confounding Factors Identified?	5. Were Strategies to Deal with Confounding Factors Stated?	6. Were the Groups/Participants Free of the Outcome at the Start of the Study (or at the Moment of Exposure)?	7. Were the Outcomes Measured in a Valid and Reliable Way?	8. Was the Follow-Up Time Reported and Sufficient to Be Long Enough for Outcomes to Occur?	9. Was Follow-Up Complete, and If Not, Were the Reasons to Loss of Follow-Up Described and Explored?	10. Were Strategies to Address Incomplete Follow-Up Utilized?	11. Was Appropriate Statistical Analysis Used?	Overall *
Bertoia et al., (2015) [33]	Y	Y	Y	Y	Y	N/A	Y	Y	Y	Y	Y	Include
Butler et al., (2004) [34]	U	Y	Y	N	N	N/A	Y	Y	N	Y	N	Exclude
Esfahani et al., (2014) [35]	Y	Y	Y	N^!!^	Y	N/A	Y	Y	Y	N	Y	Include
Halkjaer et al., (2009) [36]	Y	Y	N #	Y	Y	N/A	Y	Y	Y	N	Y	Include
He et al., (2004) [37]	Y	Y	Y	Y	Y	N/A	Y	Y	Y	N	Y	Include
Kahn et al., (1997) [38]	Y	Y	N	Y	Y	N/A	N	Y	Y	U	Y	Exclude
Koenders et al., (2011) [39]	Y	Y	N	N	N	N/A	N	Y	U	N	Y	Exclude
Mozaffarian et al., (2011) [40]	Y	Y	Y	Y	Y	N/A	Y	Y	Y	Y	Y	Include
Quick et al., (2013) [41]	Y	Y	Y	Y	Y	N/A	Y	Y	N	U	Y	Include
Rautiainen et al., (2015) [42]	Y	Y	N #	Y	Y	N/A	Y	Y	Y	U	Y	Include
Sawada et al., (2015) [43]	Y	Y	N #	Y	Y	N/A	Y	Y	Y	N	Y	Include
Souza et al., (2018) [46]	Y	Y	U #	N♦	N♦	N/A	Y	Y	Y	N	Y	Exclude
Vergnaud et al., (2012) [44]	Y	Y	N #	Y	Y	N/A	Y	Y	Y	N	Y	Include
Vioque et al., (2008) [45]	Y	Y	N ^	Y	Y	N/A	Y	Y	N	U	Y	Include

* Exclusion based on ≥3 criterion not met; # Only measured vegetable intake at baseline; ^ Adjusted for self-reported change in vegetable intake as “yes/no”, did not use validated food questionnaire at follow-up; ^!!^ Adjusted for key confounders but no adjustments made for energy intake (kJ); ♦ Adjusted for sex, follow-up time, initial BMI, and initial waist circumference, but did not adjust for physical activity or energy intake (kJ). N/A; not applicable. N: No, U: Unclear, Y: Yes.

**Table 3:** Validity of methods for dietary assessment and measure of anthropometric variables.

Author	Dietary Assessment Method for Vegetables and Unit of Measure	Method for Assessing Anthropometric Variables
Bertoia M et al., 2015 [33]	Validated FFQ [47] servings/day	Self-reported weight (lb) and height, validated in a subsample of cohort (*r* = 0.97) [48]
Butler et al., (2004) [34]	Validated Block FFQ [49], servings/day	Measured by trained technicians using Detecto balance beam scales
Esfahani et al., 2014 [35]	Validated semi-quantitative FFQ [50], servings/day	Measured by trained technicians using digital scales
Halkjaer, et al., 2009 [36]	Validated FFQ [51] potatoes separated from vegetables, only assessed at baseline, food groups converted to kilocalories/day	Baseline waist circumference and weight measured by trained technicians. Follow-up was self-measured validated in a subsample of the cohort with some degree of over/underestimation found [52].
He, et al., 2004 [37]	Validated FFQ [53], servings/day	Self-reported weight and height validated in subsample of cohort, (*r* = 0.96) [54]
Kahn et al., 1997 [38]	Self-report questionnaire 28 food items (6 vegetables) (non-validated), quintiles of intake	Self-reported weight and height (non-validated)
Koenders et al., (2011) [39]	Short question with three items (non-validated), g per day	Self-reported weight and height (non-validated)
Mozaffarian, D et al., 2011 [40]	Validated FFQs [55,56], vegetables and potatoes are separated, servings/day	Self-reported weight and height validated in subsample of cohort (*r* = 0.96) [48,57]
Quick, et al., 2013 [41]	Validated FFQ [58,59], servings/day	Self-reported height and weight, (male *r* = 0.88; female *r* = 0.85) [60].
Rautiainen, S et al., 2015 [42]	Validated FFQ [53], only assessed at baseline, servings/day	Self-reported weight and height, validated in a subsample of the cohort (*r* = 0.95 males; *r* = 0.98 females) [48]
Sawada, et al., 2015 [43]	Validated BDHQ (brief-type self-administered diet history questionnaire) at baseline only [61], g/1000 kcal/day	Weight and height measured by trained technicians
Souza et al., (2018) [46]	Frequency of food intake questionnaire (non-validated), daily frequency of intake of vegetables	Weight and height were measured using standardized scales and stadiometer
Vergnaud, et al., 2012 [44]	Validated dietary questionnaire [62] with country-specific adaptations, only assessed at baseline, g/day	Weight and height were measured at the centres using standardized procedures #
Vioque, et al., 2008 [45]	Validated FFQ [63], g/day	Weight and height measured by trained technicians

FFQ; food frequency questionnaire; BDHQ; brief-type self-administered diet history questionnaire; # Exceptions were in France, Norway, and the health-conscious group of the Oxford centre, which were self-reported.

**Table 4:** Characteristics of studies including country, population demographics, sample size, eligibility criteria, duration of study, and retention.

Author, Year, Country, Cohort	Follow-up Period (in Years), Retention %	Size of Sample, Median/Mean Age at Baseline (in Years), Gender	Eligibility Criteria of Population Included in Results
Bertoia M et al., 2015 [33] USA Nurses’ Health Study (NHS), Health Professionals Follow-Up Study (HPFS), Nurses’ Health Study II (NHS II)	Results reported per 4 year interval with a total of 6 4-year time intervals in the NHS and HPFS (1986–2010, 24 years) & four 4-year time intervals in the NHS II (1991–2007, 16 years). NHS: >90% retention, NHS II: >90% retention, HPFS: 96% retention	NHS: 35,408 women (~48.7 years) HPFS: 17,996 men (~47 years) NHS II: 64,514 women (~36.4 years)	Exclusions: history of chronic disease at baseline, gastric bypass surgery, pregnancy (one 4-year interval only), aged over 65 years old, missing data, implausible energy intake. Censored individuals who developed these conditions during follow-up: at time of diagnosis for CVD and 6 years prior for all other diseases.
Butler et al., (2004) [34] USA Female College Freshman	20 weeks Retention: 66%	*N* = 54, all women Mean age 17.79 years	Exclusions: None specified
Esfahani et al., 2014 [35] Iran Tehran Lipid and Glucose Study (TLGS)	Study used data from those measured after a 3 year time interval with baseline data collected between 2005–2008 and follow up between 2008–2011, 83% retention before exclusions	851 adults Men: 378 (mean age 40.2 years) Women: 473 (mean age 38.6 years)	Exclusions: Those who were pregnant, had cancer, stroke, or consumed drugs affecting body weight. Those with no follow-up data, under- or over reporters and those with extreme changes in weight (> 5 kg/years).
Halkjaer et al., 2009 [36] Denmark Danish Diet, Cancer, and Health Study	5.3 years (median) Retention: 83% [64]	44,897 adults Women: 22,570 (median age 56 years) Men: 20,126 (median age 55 years)	Exclusions: those registered in the Danish Cancer Registry with a previous cancer diagnosis, those who were not aged 50–64 years, were not born in Denmark or living in the greater Copenhagen or Aarhus areas
He, K et al., 2004 [37] USA, NHS	12 years >90% retention	74063 females Mean age 50.7 years (38–63 years)	Exclusions: women with history of cardiovascular disease, cancer or diabetes; or who provided incomplete or implausible information.
Kahn et al., 1997 [38] USA Cancer Prevention Study II	10 years Retention: N/A (baseline sample size not reported so retention could not be calculated)	79,236 Women: 44,080 Men: 35,156 Mean age not reported	Exclusions: those more than 54 years old at baseline, very overweight (BMI ≥ 32 kg/m^2^) or very underweight (BMI < 18 kg/m^2^) or if they reported an extreme 10-year change in BMI (increase or decrease of greater than 8 kg/m^2^. Those reporting regular use of diuretics, have a cancer history other than nonmelanoma skin cancer, diabetes, or race/ethnicity other than White non-Hispanic
Koenders et al., (2011) [39] Netherlands Workers within large banking corporation	2 years Retention: 52%	1562 Women:599 Men:963 Mean age: 44.10 years	Exclusions: None reported.
Mozaffarian et al., 2011 [40] USA NHS, NHS II, HPFS	Data based on 20 years follow-up (1986–2006) in NHS, 12 years follow-up (1991–2003) in NHS II, and 20 years follow-up (1986–2006) in HPFS. NHS: >90% retention, NHS II: >90% retention, HPFS: 96% retention	NHS: 50,422 (all women) mean age 52.2 years NHS II: 47,898 (all women) mean age 37.5 years HPFS: 22,557 (all men) mean age 50.8 years	Exclusions: participants with obesity, diabetes, cancer, or cardiovascular, pulmonary, renal or liver disease at baseline; those with missing data; those with an implausible energy intake; those who were newly pregnant during follow-up; those over 65 years
Quick et al., 2013 [41] USA, Project EAT (eating and activity in teens and young adults)	10 years 66.4% response rate	2134 participants (1133 female, 1001 male) mean age 15 years at baseline, 25.4 years at follow-up	Exclusions: those with missing data, or pregnant at follow-up.
Rautiainen et al., 2015 [42] USA, Women’s Health Study (WHS)	Mean follow-up of 15.9 years Annual questionnaires, Retention: 85%	18,146 women aged 45 or over mean age ~53.8 years	Exclusions: If diagnosed with CVD or cancer with an initial BMI less than 18.5 or greater than 25 kg/m^2^
Sawada et al., 2015 [43] Japan, Food manufacturing employees	1 year Retention N/A	478 (mean age 36.9) Aged 19–60 years	Exclusions: participants who had not received an annual health check-up or who had complete data.
Souza et al., (2018) [46] Brazil Local residents from Firminópolis town in Brzail	13.2 years Retention: 69%	1167 individuals (430 men and 737 women)	Exclusions: At follow-up were if respondent moved to another city, not found at their homes, those refusing to participate, those with physical or mental incapacity or 10 incomplete data on weight and height.
Vergnaud et al., 2012 [44] Participants from 10 European countries European Prospective Investigation into Cancer and Nutrition study (EPIC)	2–11 years Retention: 81%	373,803 (103,455 men and 270,348 women) mean age 52.7 years	Exclusions: participants with chronic disease at baseline, who were pregnant, had missing information, or those in the lowest and highest 1% of the ratio of reported total energy intake: energy requirement
Vioque et al., 2008 [45] Spain	10 years 51% response rate	206 (89 men and 117 women) Mean age 41.52 years (15–80 years)	Exclusions: those with incomplete/missing data

CVD, cardiovascular disease; BMI, Body Mass Index; N/A, not available.

**Table 5:** Summary of results and direction of impact on anthropometric outcomes.

**Study Author, Year**	**Anthropometric Measure**	**Units of Vegetable Intake**	**Results**	**Summary of Direction of Change**
Bertoia et al., ^ (2015) [33]	Weight loss (kg)	Per ↑ 1 vegetable serving/day	−0.1 kg per daily serving; 95% confidence intervals (CI) −0.35 to −0.14 Potatoes ^α^ +0.3 kg per daily serving; 95% CI 0.19 to 1.30 ^1^	Non-starchy veg = ↓ weight Potatoes = ↑ weight
Butler et al., (2004) [34]	Body weight (kg), BMI, Body Composition (% fat), Fat mass, Fat-free mass	Per ↓ 0.34 vegetable serving/day	per 0.34 ↓ in daily vegetable servings: Weight +0.72 kg (SD 0.14) BMI +0.27 kg/m^2^ (SD 0.02) % Fat +1.79 (0.24) Fat mass: +2.89 (0.38) Fat-free mass: −1.35 (0.53) *p* < 0.01 for all of above ^2^	Decreased veg intake = ↑ in weight, Body Mass Index (BMI), % fat and fat mass
Esfahani et al., (2014) [35]	Odds ratio (OR) for weight loss (kg)	Per mean ↑ of 0.2 servings/day in men and 0.3 servings/day in women	Decreased vegetable intake compared to no change, reduced the likelihood of weight loss in women by 56% (OR: 0.44, 95% CI: 0.21−0.91). MEN: no significant associations. ^3^	WOMEN: Decreased veg intake = ↓ likelihood of weight loss MEN: NS
He et al., (2004) [37] Women only	OR for risk of Obesity	Per ↑ by 1.2 vegetable servings/day	Q4 (1.2 servings) vs. Q1 (−1.72 servings) OR = 0.85; 95% CI 0.76 to 0.94(*p* trend 0.0002) ^4^	↓ risk obesity
	OR for major weight gain (>25 kg)	Per ↑2.8 vegetable servings/day	Q5 (2.8 servings) vs. Q1 (−1.72 servings) OR = 0.76; 95% CI 0.59 to 0.99 (*p* trend 0.05) ^4^	↓ risk major weight gain >25 kg
Kahn et al., 1997 [38]	Change in BMI	Lowest quintile intake compared to highest quintile	Men: −0.12 kg/m^2^ SE 0.05 (*p* = 0.012) Women: −0.12 kg/m^2^ SE 0.05 (*p* = 0.009) ^5^	↓ BMI by 0.12 kg/m^2^
	OR for weight gain at the waist		Men: OR: 0.81 95% CI 0.71, 0.93 Women: OR: 0.71 95% CI 0.59, 0.86 ^5^	↓ risk weight gain at the waist
Koenders et al., (2011) [39]	Change in BMI	Unclear	−0.045 standard error (SE): 0.055 (*p* = 0.416) ^6^	NS
Mozaffarian et al., ^ (2011) [40]	Weight loss (kg)	Per ↑ 1 vegetable serving/day	−0.09 kg per daily serving; 95% CI −0.34 to −0.11 Potatoes ^α^ +0.6 kg; 95% CI 0.87 to 1.70 (*p* < 0.001) ^7^	Non-starchy veg = ↓ weight Potatoes = ↑ weight
Souza et al., (2018) [46]	Risk of new-onset overweight/obesity	Quartiles of mean daily frequency of intake	RESULTS NOT REPORTED, only p value (*p* = 0.850) ^8^	NS
Quick et al., (2013) [41]	OR for risk of becoming overweight	Per ↑ 1 vegetable serving/day	MEN: OR = 0.88, 95% CI = 0.78 to 0.99 ^9^	MEN: ↓ risk overweight
			WOMEN: no significant associations ^9^.	WOMEN: NS
Vioque et al., (2008) [45]	OR for weight gain (>3.4 kg)	Q4 (>333 g/day) vs. Q1 (<166 g/day) adjusted baseline intakes *	Q4 vs. Q1: OR: 0.18; 95% CI 0.05 to 0.66 (*p* trend 0.032) ^10^	↓ risk weight gain >3.4 kg
**Measured Vegetable Intake at BASELINE ONLY**				
**Study Author, Year**	**Anthropometric Measure**	**Units of Vegetable Intake**	**Results**	**Summary of Direction of Change**
Halkjaer et al., (2009) [36]	Waist circumference (WC) (cm)	Per 1 vegetable serving **	WOMEN: −0.36 cm per veg serving/day (excluding potatoes) 95% CI −0.52 to −0.21, Potatoes 0.10 cm WC per serving potato/day ^#^ 95% CI: 0.006 to 0.19 ^11^	WOMEN: ↓ WC POTATOES: ↑ WC
			MEN: no significant associations with WC ^11^	MEN: NS
Rautiainen et al., (2015) [42] Women only	OR for overweight or obesity	Intake Quintile 1 (<2 servings/day) vs. Quintile 5(>5.4 servings per day)	No significant associations between vegetable intake and risk of becoming overweight or obese ^12^.	NS
Sawada et al., (2015) [43]	OR gaining >3 kg in 1 year	Intake Quartile 1(<57.2 g/1000 kcal) vs. Quartile 4 (>143.7 g/1000 kcal)	Q4 vs. Q1: OR = 0.27; 95% CI 0.08 to 0.99 (*p* trend 0.028) ^13^	↓ risk weight gain >3 kg in 1 year
Vergnaud et al., (2012) [44]	Weight loss (g)	Per ↑ vegetables by 100 g per day	MEN: −10 g; 95% CI −17 to −3; *p* = 0.007) (association disappeared with calibrated♦ data) ^14^	MEN: NS when use calibrated data
			WOMEN: No significant observations were made ^14^	WOMEN: NS

NS = Not significant, #1 potato serving approximately equal to 60 kcal/day or ½ a medium potato (not french fries); ^α^ unprocessed potatoes (baked, boiled, or mashed white potatoes, sweet potatoes, and yams); ^ use same cohort from NHS, NHSII, and HPS; ** 1 vegetable serving = 60 kcal; ↓ decrease; ↑ increase; ♦ = Calibrated data accounts for systematic and random errors in the measurement of dietary intakes between centers of the EPIC cohort; * adjusted for self-reported change in vegetable intake at 10 years measured as “yes/no”. Factors adjusted for in study analysis: 1. baseline age and BMI and change in the following lifestyle variables: smoking status, physical activity, hours of sitting or watching TV, hours of sleep, fried potatoes, juice, whole grains, refined grains, fried foods, nuts, whole-fat dairy, low-fat dairy, sugar-sweetened beverages, sweets, processed meats, non-processed meats, trans fat, alcohol, and seafood; 2. Adjustments not reported; 3. Adjustments not reported; 4. age, year of follow-up, change in physical activity, change in cigarette smoking status, changes in alcohol consumption and caffeine intake, change in use of hormone replacement therapy, and changes in energy-adjusted intakes of saturated fat, polyunsaturated fat, monounsaturated fat, trans-unsaturated fatty acid, protein, and total energy and baseline BMI; 5. age, education, body mass index in 1982, slope of body mass index between 18 years of age and 1982, change in marital status, four regions of the country, estimated total daily intake of calories in 1992, smoking, diet, and physical activity; 6. Adjustments not reported; 7. age, baseline body-mass index at the beginning of each four-year period, and sleep duration, as well as or changes in physical activity, alcohol use, television watching, smoking, and all the dietary factors; 8. sex, follow-up time, initial BMI, and waist circumference; 9. age, socioeconomic status (SES), and race/ethnicity, caloric intake, and Time 1 predictor variable; 10. sex, age, educational level, BMI, smoking habit, participation in regular activity programs, TV watching, presence of disease, hours slept per day (including afternoon naps), total energy, and energy-adjusted intakes of protein, saturated fat, monounsaturated fat, polyunsaturated, fiber, caffeine, and alcohol; 11. Baseline waist circumference, body mass index, age, smoking, sport (yes/no), hours of sport, energy intake from wine, beer, and spirits, and baseline energy intake; 12. age, smoking status, physical activity, postmenopausal status, hormone replacement therapy use, history of hypertension (yes and no), history of hypercholesterolemia (yes and no), alcohol intake, and BMI; 13. baseline age, sex, energy intake, and consumption of other foods; 14. age at recruitment and an indicator of consumption (1 = consumers and 0 = nonconsumers of fruit and vegetables, BMI at baseline, follow-up time, educational level, physical activity level, change in smoking status, total energy intake, energy intake from alcohol, and plausibility of total energy intake reporting.

**Table 6:** Overall quality assessment of nine cohort studies (796,069 participants in total) examining the impact of vegetable consumption on anthropometric outcomes using the Grading of Recommendations Assessment, Development and Evaluation (GRADE) system.

Category	Rating with Reasoning
Limitations	−1 quality levels due to limitations related to measurement of exposure
Inconsistency	No subtraction of levels, as inconsistency does not affect confidence in results
Directness of evidence	−1 level due to indirect measure of exposure over time
Precision	No subtraction of levels as the total sample size of included studies was large
Publication bias	No subtraction of levels, as studies with both significant and insignificant outcomes included and grey literature adequately searched
Upgrading factors: Dose response	+1 as 3 studies clearly indicated a dose response whereby higher vegetable intakes were associated with the lowest risks of weight gain
Overall quality	Moderate: our confidence in the overall evidence is moderate, as the true effect is likely to be close to the estimate of the effect but there is possibility that it is different

## Supplementary Materials

The following are available online at http://www.mdpi.com/2072-6643/10/11/1626/s1, Table S1: Joanna Briggs Institute Critical Appraisal Checklist for Cohort Studies—Outcomes from the two reviewers Figure S1: Forest Plot indicating the effect size calculated from odds ratios or the means and standard error for studies *n* = 7 included in the review that measured change in vegetable intake over time. Studies which measured intake at baseline only, or were considered poor quality were excluded.
