# Supplementary material for: The Relationship between Vegetable Intake and Weight Outcomes: A Systematic Review of Cohort Studies
Source: Nutrients. 2018 Nov 2;10(11):1626. doi: 10.3390/nu10111626 (PMC6266069; doi:10.3390/nu10111626)
Supplement: Supplementary file 1 [file nutrients-10-01626-s001.pdf]

Table S1: Joanna Briggs Institute Critical Appraisal Checklist for Cohort Studies – Outcomes from the two reviewers

**JBI Checklist no. Study**	1. Two groups similar and recruited from the same population?			2. Were the exposures measured similarly to assign people to both exposed and unexposed groups?			3. Was the exposure measured in a valid and reliable way?			4. Were confounding factors identified?			5. Were strategies to deal with confounding factors stated?			6. Were the groups/participants free of the outcome at the start of the study (or at the moment of exposure)?	7. Were the outcomes measured in a valid and reliable way?			8. Was the follow-up time reported and sufficient to be long enough for outcomes to occur?			9. Was follow-up complete, and if not, were the reasons to loss of follow-up described and explored?			10. Were strategies to address incomplete follow-up utilized?			11. Was appropriate statistical analysis used?			Overall*
**Reviewer decision**	1	2	**1&2**	1	2	**1&2**	1	2	**1&2**	1	2	**1&2**	1	2	**1&2**		1	2	**1&2**	1	2	**1&2**	1	2	**1&2**	1	2	**1&2**	1	2	**1&2**	
**Bertoia et al. (2015)[33]**	Y	Y	**Y**	Y	Y	**Y**	Y	Y	**Y**	Y	Y	**Y**	Y	Y	**Y**	N/A	Y	Y	**Y**	Y	Y	**Y**	Y	Y	**Y**	Y	Y	**Y**	Y	Y	**Y**	Include
**Butler et al. (2004)[34]**	Y	U	**U**	Y	Y	**Y**	Y	Y	**Y**	N	U	**N**	N	U	**N**	N/A	Y	Y	**Y**	Y	Y	**Y**	N	Y	**N**	Y	U	**Y**	N	Y	**N**	**Exclude**
**Esfahani et al. (2014)[35]**	Y	Y	**Y**	Y	Y	**Y**	Y	Y	**Y**	N	Y	**N‼**	Y	Y	**Y**	N/A	Y	Y	**Y**	Y	Y	**Y**	Y	Y	**Y**	U	N	**N**	Y	Y	**Y**	Include
**Halkjaer et al. (2009)[36]**	Y	Y	**Y**	Y	Y	**Y**	N	Y	**N#**	Y	Y	**Y**	Y	Y	**Y**	N/A	Y	Y	**Y**	Y	Y	**Y**	Y	Y	**Y**	N	N	**N**	Y	Y	**Y**	Include
**He et al. (2004)[37]**	Y	Y	**Y**	Y	Y	**Y**	Y	Y	**Y**	Y	Y	**Y**	Y	Y	**Y**	N/A	Y	Y	**Y**	Y	Y	**Y**	Y	Y	**Y**	N	N	**N**	Y	Y	**Y**	Include
**Kahn et al 1997[38]**	Y	Y	**Y**	Y	Y	**Y**	U	N	**N**	Y	Y	**Y**	U	Y	**Y**	N/A	U	N	**N**	Y	Y	**Y**	Y	Y	**Y**	U	U	**U**	Y	Y	**Y**	**Exclude**
**Koenders et al. (2011)[39]**	Y	Y	**Y**	N	Y	**Y**	N	N	**N**	N	N	**N**	N	U	**N**	N/A	N	N	**N**	Y	Y	**Y**	U	U	**U**	U	N	**N**	N	Y	**Y**	**Exclude**
**Mozaffarian et al. (2011)[40]**	Y	Y	**Y**	Y	Y	**Y**	Y	Y	**Y**	Y	Y	**Y**	Y	Y	**Y**	N/A	Y	Y	**Y**	Y	Y	**Y**	Y	U	**Y**	Y	U	**Y**	Y	Y	**Y**	Include
**Quick et al. (2013)[41]**	Y	Y	**Y**	Y	Y	**Y**	Y	Y	**Y**	Y	Y	**Y**	Y	Y	**Y**	N/A	Y	Y	**Y**	Y	Y	**Y**	N	U	**N**	Y	U	**U**	Y	Y	**Y**	Include
**Rautiainen et al. (2015)[42]**	Y	Y	**Y**	U	Y	**Y**	N	Y	**N#**	Y	Y	**Y**	Y	U	**Y**	N/A	Y	Y	**Y**	Y	Y	**Y**	Y	U	**Y**	U	U	**U**	Y	Y	**Y**	Include
**Sawada et al. (2015)[43]**	Y	Y	**Y**	U	Y	**Y**	N	Y	**N#**	Y	Y	**Y**	Y	Y	**Y**	N/A	Y	Y	**Y**	Y	Y	**Y**	U	Y	**Y**	U	Y	**N**	Y	Y	**Y**	Include
**Souza et al. (2018)[46]**	Y	Y	**Y**	Y	Y	**Y**	U	U	**U#**	N	U	**N♦**	N	U	**N♦**	N/A	Y	Y	**Y**	Y	Y	**Y**	Y	Y	**Y**	U	N	**N**	Y	Y	**Y**	**Exclude**
**Vergnaud et al. (2012)[44]**	Y	Y	**Y**	Y	Y	**Y**	N	Y	**N#**	Y	Y	**Y**	Y	Y	**Y**	N/A	Y	Y	**Y**	Y	Y	**Y**	Y	U	**Y**	N	U	**N**	Y	Y	**Y**	Include
**Vioque et al. (2008)[45]**	Y	Y	**Y**	Y	Y	**Y**	U	Y	**N^**	Y	Y	**Y**	Y	Y	**Y**	N/A	Y	Y	**Y**	Y	Y	**Y**	N	Y	**N**	U	Y	**U**	Y	U	**Y**	Include

*Exclusion based on ≥3 criterion not met; #Only measured vegetable intake at baseline; ^ Adjusted for self-reported change in vegetable intake as "yes/no", did not use validated food questionnaire at follow-up, ‼Adjusted for key confounders but no adjustments made for energy intake (kJ), ♦Adjusted for sex, follow-up time, initial BMI and initial waist circumference, but did not adjust for physical activity or energy intake (kJ). N/A; not applicable.

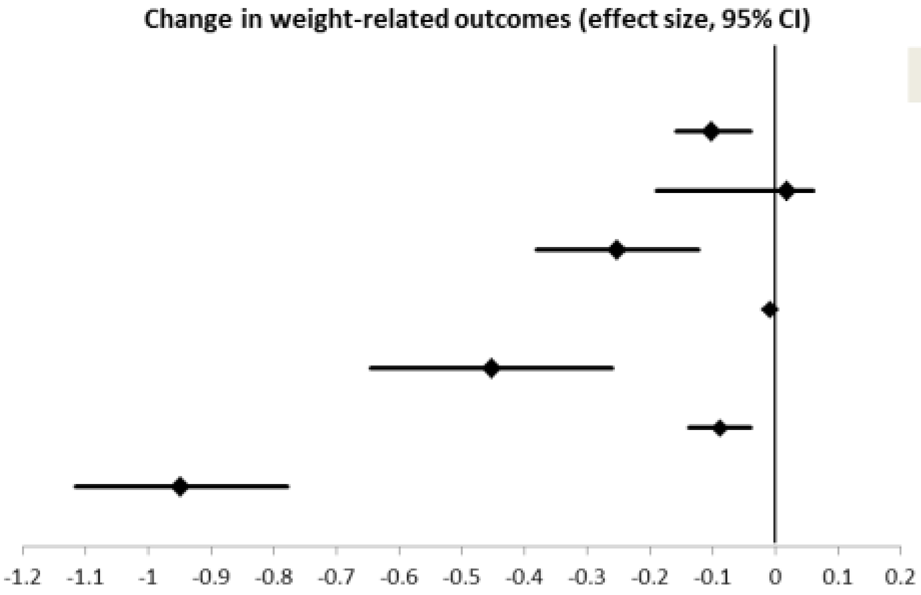

Study Author, Year	Weight-related outcome
Quick et al, 2013 (Males)	Risk of becoming overweight
Quick et al, 2013 (Females)	Risk of becoming overweight
Mozaffarian et al, 2011	Weight loss
Bertoia et al, 2015	Weight loss
Esfahani et al, 2014	Weight loss
He et al, 2004	Risk of Obesity
Vioque et al, 2008	Risk of weight gain

Figure S1: Forest Plot indicating the effect size calculated from odds ratios or the means and standard error for studies n=7 included in the review that measured change in vegetable intake over time. Studies which measured intake at baseline only, or were considered poor quality were excluded.
